# Nanopore Sequencing and *De Novo* Assembly of a Black-Shelled Pacific Oyster (*Crassostrea gigas*) Genome

**DOI:** 10.3389/fgene.2019.01211

**Published:** 2019-11-22

**Authors:** Xiaotong Wang, Wenjie Xu, Lei Wei, Chenglong Zhu, Cheng He, Hongce Song, Zhongqiang Cai, Wenchao Yu, Qiuyun Jiang, Lingling Li, Kun Wang, Chenguang Feng

**Affiliations:** ^1^School of Agriculture, Ludong University, Yantai, China; ^2^Center for Ecological and Environmental Sciences, Northwestern Polytechnical University, Xi’an, China; ^3^Changdao Enhancement and Experiment Station, Chinese Academy of Fishery Sciences, Changdao, China

**Keywords:** *Crassostrea gigas*, black-shelled pacific oyster, evolution, genome assembly, nanopore sequencing

## Abstract

The Pacific oyster, *Crassostrea gigas*, belongs to one of the most species-rich phyla and provides important ecological and economical services. Here we present a genome assembly for a variety of this species, black-shelled Pacific oyster, using a combination of 61.8 Gb Nanopore long reads and 105.6 Gb raw BGI-seq short reads. The genome assembly comprised 3,676 contigs, with a total length of 587 Mb and a contig N50 of 581 kb. Annotation of the genome assembly identified 283 Mb (48.32%) of repetitive sequences and a total of 26,811 protein-coding genes. A long-term transposable element active, accompanied by recent expansion (1 million years ago), was detected in this genome. The divergence between black-shelled and the previous published Pacific oysters was estimated at about 2.2 million years ago, which implies that species *C. gigas* had great intraspecific genetic variations. Moreover, we identified 148/188 specifically expanded/contracted gene families in this genome. We believe this genome assembly will be a valuable resource for understanding the genetic breeding, conservation, and evolution of oysters and bivalves.

## Introduction

Fossil records show that oysters appeared about 200 million years ago (Upper Triassic; Stott, 2004). From then on, they began their work of filtering the oceans. Because of their “advanced” defense mechanism (thick shells), which confers strong resistance to desiccation and sunlight exposure, they underwent a large increase during the following 70 million years (from the Jurassic to the Cretaceous). By about 135 million years ago, oysters were the predominant shellfish in the world’s ocean (Stott, 2004). Following settlement and metamorphosis, oysters attach their left shells to other objects and begin a sessile lifestyle (Zhang et al., 2012). Individuals of all ages cluster together and eventually form reefs, which harbor many kinds of marine organisms. Thus, the oyster is of great significance to marine ecology (Beck et al., 2011).

Because oysters are sessile animals living in estuarine and intertidal regions, they have to cope with drastically changing environments. Hence, they are ideal models for investigating adaptations of living organisms to climate change (Li et al., 2018). In addition, oysters are frequently used as models in studies on neurobiology, biomineralization, ocean acidification, immunity, and developmental biology (Caldeira and Wickett, 2003).

The Pacific oyster, *Crassostrea gigas* (Bivalvia, Osteroida, Ostreidae), is economically important in aquaculture around the world (Zhang et al., 2016). Several famous types of oysters, such as the Gillardeau oyster from France and the Rock Oyster from Australia, are Pacific oysters. Because they are of great value to the economy, scientific research, and ecology, Pacific oysters have received extensive attention (Wrange et al., 2010; Du et al., 2017; Sun et al., 2017; De Lorgeril et al., 2018; Powell et al., 2018). Moreover, a number of varieties of this species have been bred, such as the black-shelled Pacific oyster (BPO; Hao et al., 2015). Prior to this work, a Pacific oyster genome sequence had been reported in 2012 (Zhang et al., 2012). However, the extensive intraspecific variations that characterize the Pacific oyster (Li et al., 2018) mean that the previous genome has been of limited assistance to oyster research and the oyster industry. Hence, there is a pressing need for high-quality genome maps of multiple varieties.

Here, we present a high-quality genome assembly for the BPO bred by our lab, constructed using both Nanopore long reads and BGI-seq short reads. Some aspects of this assembly are superior to those of the earlier one mainly due to much advanced technologies. We think that this well-annotated genome assembly and the massive amount of sequencing data generated in this study will serve as substantial resources for future oyster academic research and industrial development.

## Materials and Methods

### Sample Collection, Library Construction, and Sequencing

**Figure 1:**
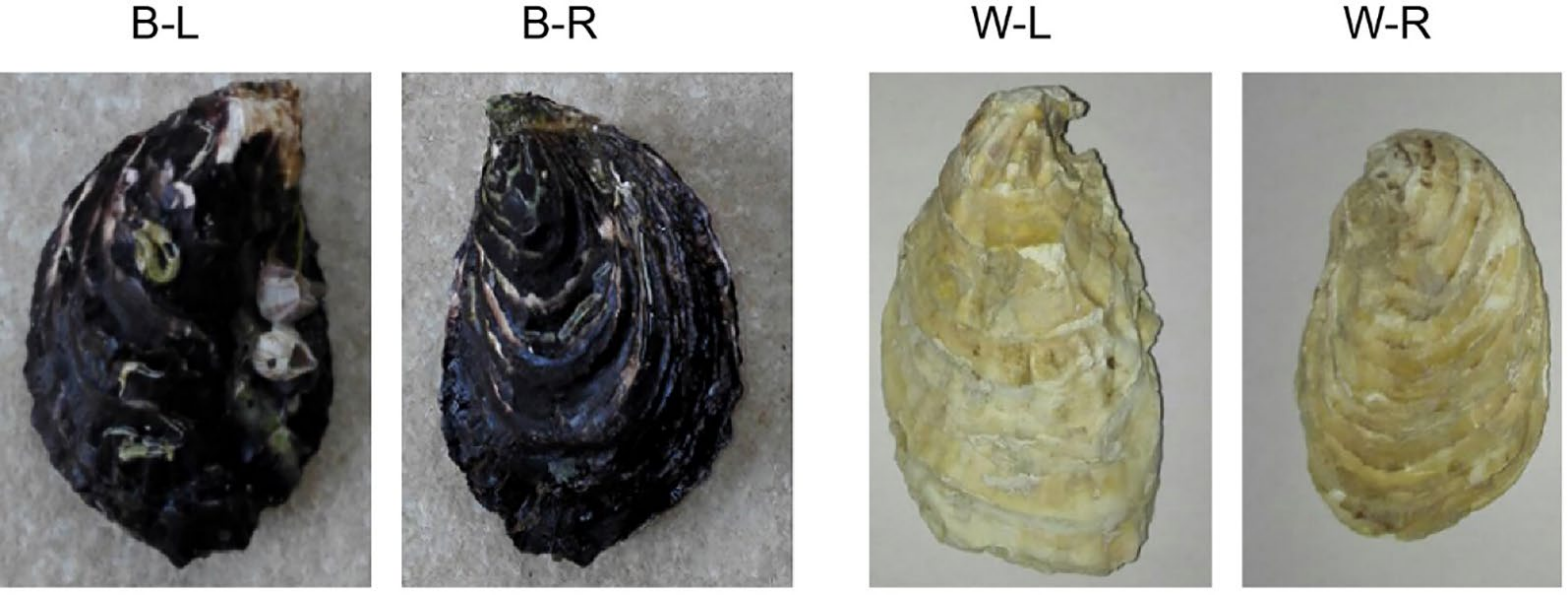
A photograph of black-shelled and white-shelled Pacific oysters, *Crassostrea gigas*. B-L denotes the left shell of a black-shelled oyster; B-R denotes the right shell of a black-shelled oyster; W-L denotes the left shell of a white-shelled oyster; W-R denotes the right shell of a white-shelled oyster.

An individual of the BPO (*C. gigas*, NCBI taxonomy ID: 29159; **Figure 1**), collected from Changdao County, Yantai City, Shandong Province, China, was used for whole-genome sequencing. Genomic DNA was isolated from the mantles using a Qiagen Blood & Cell Culture DNA Mini Kit according to the manufacturer’s instructions. A BGI-seq library was constructed using a MGIEasy Library Prep Kit V1.1 (MGI Tech), and paired-end 150 bp single-indexed sequencing was performed on the MGISEQ-2000 platform (BGI, Shenzhen, China; Winnepenninckx et al., 1993). Nanopore libraries were prepared and sequenced on two flow-cells using a GridION DNA sequencer according to the manufacturer’s instructions (Oxford Nanopore, Oxford, UK; Jain et al., 2016; Jain et al., 2018; Gong et al., 2019).

### Data Filtering and Genome Size Estimation

Both Nanopore and BGI-seq reads were used to achieve a high-quality genome assembly. Before assembly, these two kinds of reads were filtered as follows: For the Nanopore data, reads with mean quality scores >7 were retained and further corrected with NextDenovo (https://github.com/Nextomics/NextDenovo). For the BGI-seq data, adaptor sequences and low-quality reads were filtered out using the program fastp (v 0.20; Chen et al., 2018). Any reads with more than 30 low-quality bases or 5% unknown bases were abandoned. These reads were used for further assembly and subsequent analyses.

The genome size (G) of this Pacific oyster was estimated from k-mer (k = 23 in this case) frequency distribution analysis using the clean BGI-seq data. The term k-mer is used to refer to each of the possible successive subsequences of length k in a read. If the length of each k-mer is 23 bp, a filtered read that is L bp in length contains (L-23+1) k-mers. The genome size is estimated by the formula: G = K_num/K_depth, where K_num and K_depth are the total number and the peak frequency of 23-mers respectively (Koren et al., 2012; Ross et al., 2013; Wences and Schatz, 2015; Vurture et al., 2017).

### Genome Assembly

The corrected Nanopore reads were assembled into contigs by wtdbg (v 2.2; Ruan and Li, 2019) with the parameter “-x corrected”. After that, the filtered BGI-seq reads were mapped to the contigs using Burrows-Wheeler Aligner (BWA, RRID: SCR_010910; Li and Durbin, 2010) and then subjected to two rounds of polishing with Pilon (v 1.21; Walker et al., 2014). Finally, we employed Purge Haplotigs (v 1.0.4; Roach et al., 2018) to resolve redundancy in the assembly based on Nanopore reads depth information.

The accuracy of the genome assembly was evaluated by mapping the clean BGI-seq short reads against the genome using BWA (Li and Durbin, 2010). Furthermore, we employed Benchmarking Universal Single-Copy Orthologs (BUSCO, v 3.0.2; Simao et al., 2015), a software package that can quantitatively measure completeness of genome assembly based on evolutionarily informed expectations of gene content, to evaluate the completeness of the genome assembly, using 978 genes that are expected to be present in all metazoans (Cai et al., 2019).

### Annotation of Genomic Repeats

Both *de novo* and homology-based predictions were used to identify repetitive elements in the *C. gigas* genome. Firstly, we constructed a *de novo* repeat library using RepeatModeler (v 1.0.11; Tarailo-Graovac and Chen, 2009) and LTR_FINDER (Xu and Wang, 2007). We then used RepeatMasker (v 2.1; setting -nolow -norna -no_is; Tarailo-Graovac and Chen, 2009) to detect and classify repeats in the sequences. To search for tandem repeats, we subjected the draft genome to Tandem Repeats Finder (v 4.07; Benson, 1999), setting the following parameters: Match = 2, Mismatch = 7, Delta = 7, PM = 80, PI = 10, Minscore = 50, MaxPeriod = 500. We also annotated transposable elements (TEs) by running RepeatMasker v 2.1 and RepeatProteinMask v 2.1 (a package in RepeatMasker, with the parameters -engine ncbi -noLowSimple -pvalue 1e-04; Tarailo-Graovac and Chen, 2009) against the Repbase and TE protein databases. Prior to gene prediction, all regions of repetitive elements were soft-masked with RepeatMasker.

### Gene Prediction and Annotation

We combined *de novo* and homology-based predictions to identify protein-coding genes. For homology-based annotation, three genomes of Pteriomorphia species were downloaded from NCBI: *C. gigas* (GCA_000297895.1), *Crassostrea virginica* (GCA_002022765.4), and *Mizuhopecten yessoensis* (GCA_002113885.2). We picked the longest transcript of each gene and removed those with premature terminations. The remaining genes were translated into proteins and aligned to our genome with TBLASTN (v 2.7.1; Altschul et al., 1990) to search for the approximate positions of potential gene homologs. Next, we used Exonerate (v 2.2; Slater and Birney, 2005) to obtain an accurate gene structure for each locus. For *de novo* annotation, we first picked 898 complete genes in order to obtain parameters suitable for *C. gigas*. Then we performed *de novo* prediction on the repeat-masked genome using AUGUSTUS (v 3.2.1; Stanke et al., 2008) with the gene parameters so obtained. Finally, we used EVidenceModeler (v 1.1.1; Haas et al., 2008) to integrate homologs and *de novo* predicted genes and generate a comprehensive, non-redundant gene set. The completeness of the genome annotation was investigated using BUSCO (v 3.0.2; Simao et al., 2015) with the parameter “-l metazoa _odb9”.

We used InterProScan (v 5.30-69.0; Jones et al., 2014) with default parameters to annotate the functions of detected motifs and domains by searching public databases (GO, INTERPRO, PFAM, KEGG, and PANTHER).

### Phylogenetic Analysis and Divergence Time Estimation

Genomes of 10 other species, *Helobdella robusta* (GCA_000326865.1), *Lingula anatine* (GCA_001039355.2), *Octopus bimaculoides* (GCA_001194135.1), *Pomacea canaliculate* (GCA_003073045.1), *Lottia gigantea* (GCA_000327385.1), *Elysia chlorotica* (GCA_003991915.1), *Aplysia californica* (GCA_000002075.2), *Limnoperna fortune* (GCA_003130415.1), *Bathymodiolus platifrons* (GCA_002080005.1), and *Modiolus philippinarum* (GCA_002080025.1) were downloaded from NCBI and processed in the same way as those three species above. Then, the detected BPO genes along with those of 13 species were clustered in families by OrthoFinder (v 2.3.1; Emms and Kelly, 2015) with default parameters in an all-to-all BLASTP analysis. Subsequently, one to one orthologs were identified from these species. The sequences were aligned using MUSCLE (v 3.8.1551; Edgar, 2004) and trimmed by TrimAl (Capella-Gutiérrez et al., 2009) with algorithm automated1. The remained sequences were then concatenated into a supergene and used to construct a phylogenetic tree by RAxML (v 8.2.12; Stamatakis, 2014), with the GTRGAMMA model and 100 pseudoreplicates. Finally, the species divergence time was estimated along the ML phylogenetic tree by MCMCtree in PAML (v 4.9; Yang, 2007). Four time-calibrated points were used based on fossil records, the first appearance of Lophotrochozoa (550.3–636.1 Ma; Benson et al., 2015), the first appearance of Molluscs (532–549 Ma; Benton et al., 2015), *L. gigantea*–*A. californica* (470.2–531.5 Ma; Benson et al., 2009), and *C. gigas*–*C. virginica* (63.2–82.7 Ma; Plazzi and Passamonti, 2010; Ren et al., 2010).

We further used parseRM.pl (a custom Perl script, available at https://github.com/4ureliek/Parsing-RepeatMasker-Outputs ; Kapusta et al., 2017) to rebuild the TE accumulation. The parseRM.pl allowed us to parse the age category of each TE copy based on alignment outputs from RepeatMasker (Tarailo-Graovac and Chen, 2009). The mutation rate was set at 0.0084 per million years, which was reestimated by r8s (v 1.81; Sanderson, 2003) with the penalized likelihood method. The analysis result was packed into bin per 0.1 Ma.

### Gene Family Expansions and Contractions

We used computational analysis of gene family Evolution (CAFÉ; V 4.0.1; De Bie et al., 2006) with default settings to identify the expanded and contracted gene families, along the timetree created in the previous step. any gene family under both family-wide and viterbi *P*-Values < 0.01 was retained. Then we conducted GO enrichment analysis to investigate functional categories of the expanded and contracted gene families under the standard of Chi.FDR < 0.05 (Xiao et al., 2018).

## Results and Discussions

In total, 105.6 Gb of raw BGI-seq data and 61.8 Gb of Nanopore reads were produced. After the removal of low-quality reads, we harvested 104.9 Gb of clean BGI-seq reads, and 39.9 Gb of corrected Nanopore reads with a mean subread length of 21.9 kb (**Table S1**). For the 23-mer analysis, K_num was 83,754,003,275 and K_depth was estimated as 141, giving an estimated genome size of *c.* 594 Mb (**Table S2**). This genome size is within the range of 545–637 Mb reported by (Zhang et al., 2012). A bimodal pattern was observed in the 23-mer frequency distribution analysis (**Figure S1**). The heterozygous peak (the first peak) was much higher than the second, homozygous peak, indicating that the BPO had a diploid genome with a high level of heterozygosity.

Initial assembly yielded a total length of 656 Mb, comprising 6,815 contigs with a contig N50 length of 436 kb (**Table S3**). The genome assembly was larger than the estimated genome size of *c.* 594 Mb (see above), because some allelic variants failed to be merged due to high heterozygosity (Zhang et al., 2012). After eliminating the redundancy, we obtained a final genome assembly of 587 Mb for the BPO, which was pretty close to the estimated genome size, comprising 3,676 contigs with a contig N50 length of 581 kb (**Table 1** and **S3**).

**Table 1:** Assembly statistics for the genome of *Crassostrea gigas* black-shelled.

	Contigs	
	Length (bp)	Number
N90	66,669	1,570
N80	124,267	922
N70	215,826	561
N60	361,254	350
N50	581,941	220
Shortest	3,241	—
Longest	6,082,460	—
Total Size	587,503,506	3,676
Total Number (>10 Kbp)	—	3,293
Total Number (>100 Kbp)	—	1,127
Total Number (>1 Mbp)	—	109

BUSCO analysis showed that 919 (94.0%) of 978 metazoan BUSCOs were detected in the BPO genome assembly, with 890 (91.0%) and 29 (3.0%) being identified as complete and fragmented respectively (**Table 2**). Mapping rate test suggested that more than 97.42% of the clean BGI-seq reads were properly mapped to the genome, and of these 91.42% mapped to their mates. These reads covered 98.08% of the genome assembly (**Table S4**). These analyses indicated that the assembly was high quality in both levels of completeness and accuracy.

**Table 2:** Assessments of the genome and gene set using BUSCO (metazoa odb9).

Mode	Genome		Genes	
**Number**	**Number**	**Percentage (%)**	**Number**	**Percentage (%)**
Complete	890	91.0	894	91.4
Single-copy complete	881	90.1	879	89.9
Duplicated complete	9	0.9	15	1.5
Fragmented	29	3.0	34	3.5
Missing	59	6.0	50	5.1
Total	978	–	978	–

**Figure 2:**
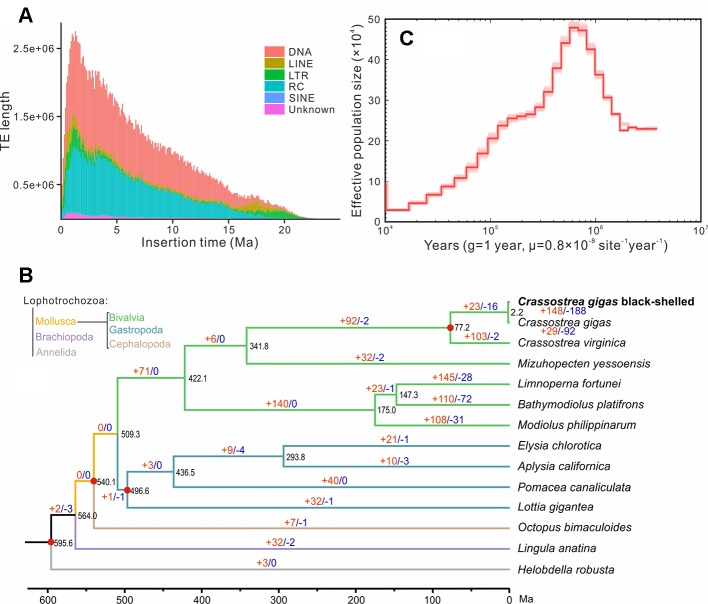
Genome evolution of the black-shelled Pacific oyster. **(A)** the whole landscape of TEs accumulation. **(B)** Phylogenetic relationships, divergence time, and expanded and contracted gene families of 14 Lophotrochozoa species. The phylogenetic tree was derived from RaxML analysis, with all nodes having a bootstrap value greater than 95. Red dots indicated time-calibration markers. Brown and blue numbers on branches represented the mount of expanded or contracted gene families, respectively. **(C)** Demographic history of the black-shelled Pacific oyster constructed by the PSMC model. g means generation. Abbreviations: TE, transposable element; DNA, DNA transposons; LINE, long interspersed nuclear element; LTR, long terminal repeat element; RC, rolling circle; SINE, short interspersed nuclear element; Ma, million years ago.

Repeat annotation identified 283 Mb (48.32%) of repetitive sequences in the assembled genome. The proportion of repetitive sequences was a bit higher than that of the previous *C. gigas* genome (36.1%; Zhang et al., 2012). DNA transposon was the most abundant repeat and accounted for 21.28% (125 Mb in total) of the genome (**Tables S5** and **S6**), while it was only 4.1% of the previous *C. gigas* genome (Zhang et al., 2012). TE accumulation analysis suggested long-term TE actives, accompanied by recent expansion within 1 million years ago (Ma), in this genome (**Figure 2A** and **Figure S2**). DNA transposons and rolling circles (RC) presented landscapes similar to the whole, whereas long terminal repeat elements (LTR) and long interspersed nuclear elements (LINE) showed ancient retention and recent expansion of the TE insertions (**Figure S2**). The BPO genome exhibits a remarkable level of recent TE actives that differed from the previous version (Zhang et al., 2012). The specific TEs accumulation may be an important reason underlying the difference in repeats repertoire between these two *C. gigas* genomes. In addition, 26,811 protein-coding genes were identified, averaging 7.0 exons and a 1,225 bp coding region per gene, which was comparable to the other Pacific oyster genome published in 2012 (**Figure S3** and **Table S7**; Zhang et al., 2012). 23,111 genes (86.2% of the predicted genes) were successfully annotated with predicted functions or conserved functional motifs. Respectively, 11,810, 14,400, 16,853, 7,575, and 15,997 genes showed positive hits in GO, PFAM, INTERPRO, KEGG, and PANTHER (**Table S8**). BUSCO (Simao et al., 2015) analysis suggested that 94.9% (928) of the metazoa core conserved genes were detected in the BPO gene set, with 894 (91.4%) and 34 (3.5%) being identified as complete and fragmented, respectively (**Table 2**). BUSCO analysis showed that the performance in the genome is better than the genome assembly, which was presumably most that it is simpler to search for genes in a transcriptome or proteome than in a genome (Cai et al., 2019).

OrthoFinder analysis identified 199 single-copy orthologues. RaxML analysis supported that the BPO was clustered with the other *C. gigas* in the phylogenetic tree. Based on four solid time-calibrations (see *MATERIALS AND METHODS*), molecular clock analysis suggests that they diverged at about 2.2 million years ago (**Figure 2B**). Previous phylogenetic studies and fossil records (http://fossilworks.org/bridge.pl?a = taxonInfo&taxon_no = 109465) indicated that species divergence between *C. gigas* and *C. virginica* was at 63.2–82.7 Ma (Plazzi and Passamonti, 2010; Ren et al., 2010). Thus, the divergence time between the BPO in this study and the other *C. gigas* sequenced in 2012 should be rational but still very long. The deep divergence, combined with remarkable difference in repeats repertoire, suggested that *C. gigas* comprised great intraspecific genetic variations. Our result was consistent with studies of Li et al. (2018), who found unexpected genetic divergence in several populations of *C. gigas*. Estimated effective population size (*Ne*) dynamic indicated that the BPO reached a top *Ne* at around 0.6 Ma, and then experienced a sustained recession (**Figure 2C**). In the end, 148 expanded gene families and 188 contracted gene families were identified in the BPO. The expanded gene families were enriched in 22 GO categories about enzymatic activity, cytoskeleton, membranes, ion transport, etc. (for details, see **Table S9**). The contracted gene families were enriched in 31 GO categories: peroxidase activity, response to oxidative stress, response to stress, and 28 others (for details, see **Table S10**). It was possible that TE activities after divergence affected the expansion and contraction of gene families (Joly-Lopez et al., 2012; Kapusta et al., 2017). However, whether these notable changes in BPO tell about special adaptations needs further exploration.

Nanopore sequencing produces long read lengths, which can effectively improve the quality of genome assembly and alleviate assembly errors (Xiao et al., 2017). The *C. gigas* genome, known as its high heterozygosity and high repetition rate, once was a challenging project in the age of Next-generation sequencing (Zhang et al., 2012). Current assembly (contig N50 of 581.9 kb) using Nanopore sequencing is superior to the previous version (contig N50 of 19.4 kb and scaffold N50 of 401 kb, Zhang et al., 2012) in genome continuity. Accuracy and integrity of this genome are also comparable to that of the previous genome. Although, reads produced by this technology have high error rates (Liu et al., 2019), several rounds of polishing before and after assembly can effectively eliminate the negative effects of sequencing errors.

## Conclusions

Here, we present high-quality genome assembly and annotation of a variety of Pacific oyster, the BPO. The deep divergence of history, specific TE repertoire, and significant expansion and contraction of gene families in this genome suggested that BPO also represents a special Pacific oyster in genetics. Hence, the BPO genome and the massive amount of data created in this study will serve as valuable resources for studying the genetic breeding, conservation, and evolution in oysters. A series of better assembly parameters suggested that nanopore sequencing technology is qualified for the assembly of complex genomes like oysters.

## Data Availability Statement

Raw reads from BGI-seq and Nanopore sequencing had been deposited in the NCBI Sequence Read Archive (SRA) database under accession number SRP193912 and BioProject accession PRJNA534417. This Whole Genome Shotgun project has been deposited at DDBJ/ENA/GenBank under the accession SZQM00000000. The version described in this paper is version SZQM02000000.

## Author Contributions

XW and CF designed and supervised the project. XW, LW, CH, HS, ZC, WY, QJ, and LL prepared the samples. WX, XW, CZ, and CF analyzed the data. WX, XW, and LW wrote the manuscript with the help of the other authors. KW and CF revised the manuscript. All authors read and approved the final manuscript.

## Funding

This study was supported by National Key R&D Program of China (2018YFD0901400), the National Natural Science Foundation of China (41876193 and 41906088), the Fundamental Research Funds for the Central Universities (19SH0304), the Key R & D Program of Shandong Province, China (2018GHY115027), the Special Funds for Taishan Scholars Project of Shandong Province, China (tsqn201812094), the Shandong Provincial Natural Science Foundation, China (ZR2019MC002), the Fine Agricultural Breeds Project of Shandong Province, China (2019LZGC020), the Modern Agricultural Industry Technology System of Shandong Province, China (SDAIT-14-03), the Key R&D Program of Yantai City, China (2017ZH054), the Plan of Excellent Youth Innovation Team of Colleges and universities in Shandong Province, China (2019KJF004) and the Research Funds for Interdisciplinary subject, NWPU (19SH030408). We gave our thanks to Dr. Feng Shao for his kind help in TE analysis.

## Conflict of Interest

The authors declare that the research was conducted in the absence of any commercial or financial relationships that could be construed as a potential conflict of interest.

## Abbreviations

BPO, Black-shelled Pacific oyster; BUSCO, Benchmarking Universal Single-Copy Orthologs; BWA, Burrows-Wheeler Aligner; CAFÉ, Computational Analysis of gene Family Evolution; FDR, False discovery rate; GO, Gene Ontology; LINE, Long interspersed nuclear element; LTR, Long terminal repeat element; ML, Maximum likelihood; Ma, Million years ago; *Ne*, Estimated effective population size; RC, Rolling circle; SINE, Short interspersed nuclear element; SRA, Sequence Read Archive; TE, Transposable element.

## References

[B1] Altschul S. F. Gish W. Miller W. Myers E. W. Lipman D. J. (1990). Basic local alignment search tool. J. Mol. Biol. 215, 403–410. 10.1016/S0022-2836(05)80360-2 2231712

[B2] Beck M. W. Brumbaugh R. D. Airoldi L. Carranza A. Coen L. D. Crawford C. (2011). Oyster reefs at risk and recommendations for conservation, restoration, and management. Bioscience 61, 107–116. 10.1525/bio.2011.61.2.5

[B3] Benson M. J. Donoghue P. C. Asher R. J. (2009). “Calibrating and constraining molecular clocks,” in The Timetree of Life. Ed. Blair Hedges S. Kumar S. (Oxford, UK:Oxford University Press), 35–86.

[B4] Benson M. J. Donoghue P. C. Asher R. J. Friedman M. Near T. J. Vinther J. (2015). Constraints on the timescale of animal evolutionary history. Palaeontologia Electronica 18 (1), 1–106. 10.26879/424

[B5] Benson G. (1999). Tandem repeats finder: a program to analyze DNA sequences. Nucleic Acids Res. 27, 573–580. 10.1093/nar/27.2.573 9862982 PMC148217

[B6] Cai H. Li Q. Fang X. Li J. Curtis N. E. Altenburger A. (2019). A draft genome assembly of the solar-powered sea slug Elysia chlorotica. Sci. Data 6, 190022. 10.1038/sdata.2019.22 30778257 PMC6380222

[B7] Caldeira K. Wickett M. E. (2003). Oceanography: anthropogenic carbon and ocean pH. Nature 425, 365. 10.1038/425365a 14508477

[B8] Capella-Gutierrez S. Silla-Martinez J. M. Gabaldon T. (2009). trimAl: a tool for automated alignment trimming in large-scale phylogenetic analyses. Bioinformatics 25 (15), 1972–1973. 10.1093/bioinformatics/btp348 19505945 PMC2712344

[B9] Chen S. Zhou Y. Chen Y. Gu J. (2018). fastp: an ultra-fast all-in-one FASTQ preprocessor. Bioinformatics 34, i884–i890. 10.1093/bioinformatics/bty560 30423086 PMC6129281

[B10] De Bie T. Cristianini N. Demuth J. P. Hahn M. W. (2006). CAFE: a computational tool for the study of gene family evolution. Bioinformatics 22, 1269–1271. 10.1093/bioinformatics/btl097 16543274

[B11] De Lorgeril J. Lucasson A. Petton B. Toulza E. Montagnani C. Clerissi C. (2018). Immune-suppression by OsHV-1 viral infection causes fatal bacteraemia in Pacific oysters. Nat. Commun. 9, 4215. 10.1038/s41467-018-06659-3 30310074 PMC6182001

[B12] Du X. Fan G. Jiao Y. Zhang H. Guo X. Huang R. (2017). The pearl oyster Pinctada fucata martensii genome and multi-omic analyses provide insights into biomineralization. GigaScience 6, gix059. 10.1093/gigascience/gix059 PMC5597905 28873964

[B13] Edgar R. C. (2004). MUSCLE: multiple sequence alignment with high accuracy and high throughput. Nucleic Acids Res. 32, 1792–1797. 10.1093/nar/gkh340 15034147 PMC390337

[B14] Emms D. M. Kelly S. (2015). OrthoFinder: solving fundamental biases in whole genome comparisons dramatically improves orthogroup inference accuracy. Genome Biol. 16, 157. 10.1186/s13059-015-0721-2 26243257 PMC4531804

[B15] Gong L. Fan G. Ren Y. Chen Y. Qiu Q. Liu L. (2019). Chromosomal level reference genome of Tachypleus tridentatus provides insights into evolution and adaptation of horseshoe crabs. Mol. Ecol. Resour. 19, 744–756. 10.1111/1755-0998.12988 30592378

[B16] Haas B. J. Salzberg S. L. Zhu W. Pertea M. Allen J. E. Orvis J. (2008). Automated eukaryotic gene structure annotation using EVidenceModeler and the Program to Assemble Spliced Alignments. Genome Biol. 9, R7. 10.1186/gb-2008-9-1-r7 18190707 PMC2395244

[B17] Hao S. Hou X. Wei L. Li J. Li Z. Wang X. (2015). Extraction and Identification of the Pigment in the Adductor Muscle Scar of Pacific Oyster Crassostrea gigas. PloS One 10, e0142439. 10.1371/journal.pone.0142439 26555720 PMC4640808

[B18] Jain M. Olsen H. E. Paten B. Akeson M. (2016). The Oxford Nanopore MinION: delivery of nanopore sequencing to the genomics community. Genome Biol. 17, 239. 10.1186/s13059-016-1103-0 27887629 PMC5124260

[B19] Jain M. Koren S. Miga K. H. Quick J. Rand A. C. Sasani T. A. (2018). Nanopore sequencing and assembly of a human genome with ultra-long reads. Nat. Biotechnol. 36, 338–345. 10.1101/128835 29431738 PMC5889714

[B20] Joly-Lopez Z. Forczek E. Hoen D. R. Juretic N. Bureau T. E. (2012). A gene family derived from transposable elements during early angiosperm evolution has reproductive fitness benefits in Arabidopsis thaliana. PloS Genet. 8, e1002931. 10.1371/journal.pgen.1002931 22969437 PMC3435246

[B21] Jones P. Binns D. Chang H. Y. Fraser M. Li W. Mcanulla C. (2014). InterProScan 5: genome-scale protein function classification. Bioinformatics 30, 1236–1240. 10.1093/bioinformatics/btu031 24451626 PMC3998142

[B22] Kapusta A. Suh A. Feschotte C. (2017). Dynamics of genome size evolution in birds and mammals. Proc. Natl. Acad. Sci. 114, E1460–E1469. 10.1073/pnas.1616702114 28179571 PMC5338432

[B23] Koren S. Schatz M. C. Walenz B. P. Martin J. Howard J. T. Ganapathy G. (2012). Hybrid error correction and de novo assembly of single-molecule sequencing reads. Nat. Biotechnol. 30, 693–700. 10.1038/nbt.2280 22750884 PMC3707490

[B24] Li H. Durbin R. (2010). Fast and accurate long-read alignment with Burrows-Wheeler transform. Bioinformatics 26, 589–595. 10.1093/bioinformatics/btp698 20080505 PMC2828108

[B25] Li L. Li A. Song K. Meng J. Guo X. Li S. (2018). Divergence and plasticity shape adaptive potential of the Pacific oyster. Nat. Ecol. Evol. 2, 1751–1760. 10.1038/s41559-018-0668-2 30250157

[B26] Liu Q. Fang L. Yu G. Wang D. Xiao C. L. Wang K. (2019). Detection of DNA base modifications by deep recurrent neural network on Oxford Nanopore sequencing data. Nat. Commun. 101, 2449. 10.1038/s41467-019-10168-2 PMC6547721 31164644

[B27] Plazzi F. Passamonti M. (2010). Towards a molecular phylogeny of Mollusks: Bivalves’ early evolution as revealed by mitochondrial genes. Mol. Phylogenet. Evol. 57, 641–657. 10.1016/j.ympev.2010.08.032 20817110

[B28] Powell D. Subramanian S. Suwansa-Ard S. Zhao M. O'connor W. Raftos D. (2018). The genome of the oyster Saccostrea offers insight into the environmental resilience of bivalves. DNA Res. 25, 655–665. 10.1093/dnares/dsy032 30295708 PMC6289776

[B29] Ren J. Liu X. Jiang F. Guo X. Liu B. (2010). Unusual conservation of mitochondrial gene order in Crassostrea oysters: evidence for recent speciation in Asia. BMC Evol. Biol. 10, 394. 10.1186/1471-2148-10-394 21189147 PMC3040558

[B30] Roach M. J. Schmidt S. A. Borneman A. R. (2018). Purge Haplotigs: allelic contig reassignment for third-gen diploid genome assemblies. BMC Bioinf. 19, 460. 10.1186/s12859-018-2485-7 PMC6267036 30497373

[B31] Ross M. G. Russ C. Costello M. Hollinger A. Lennon N. J. Hegarty R. (2013). Characterizing and measuring bias in sequence data. Genome Biol. 14, R51. 10.1186/gb-2013-14-5-r51 23718773 PMC4053816

[B32] Ruan J. Li H. (2019). Fast and accurate long-read assembly with wtdbg2. BioRxiv, 530972. 10.1101/530972 PMC7004874 31819265

[B33] Sanderson M. J. (2003). r8s: inferring absolute rates of molecular evolution and divergence times in the absence of a molecular clock. Bioinformatics 19, 301–302. 10.1093/bioinformatics/19.2.301 12538260

[B34] Simao F. A. Waterhouse R. M. Ioannidis P. Kriventseva E. V. Zdobnov E. M. (2015). BUSCO: assessing genome assembly and annotation completeness with single-copy orthologs. Bioinformatics 31, 3210–3212. 10.1093/bioinformatics/btv351 26059717

[B35] Slater G. S. Birney E. (2005). Automated generation of heuristics for biological sequence comparison. BMC Bioinf. 6, 31. 10.1186/1471-2105-6-31 PMC553969 15713233

[B36] Stamatakis A. (2014). RAxML version 8: a tool for phylogenetic analysis and post-analysis of large phylogenies. Bioinformatics 30, 1312–1313. 10.1093/bioinformatics/btu033 24451623 PMC3998144

[B37] Stanke M. Diekhans M. Baertsch R. Haussler D. (2008). Using native and syntenically mapped cDNA alignments to improve de novo gene finding. Bioinformatics 24, 637–644. 10.1093/bioinformatics/btn013 18218656

[B38] Stott R. (2004). Oyster, London:Reaktion Books LTD.

[B39] Sun J. Zhang Y. Xu T. Zhang Y. Mu H. Zhang Y. (2017). Adaptation to deep-sea chemosynthetic environments as revealed by mussel genomes. Nat. Ecol. Evol. 1, 121. 10.1038/s41559-017-0121 28812709

[B40] Sun J. Mu H. Ip J. C. H. Li R. Xu T. Accorsi A. (2019). Signatures of Divergence, Invasiveness, and Terrestrialization Revealed by Four Apple Snail Genomes. Mol. Biol. Evol. 36, 1507–1520. 10.1093/molbev/msz084 30980073 PMC6573481

[B41] Tarailo-Graovac M. Chen N. (2009). Using RepeatMasker to identify repetitive elements in genomic sequences. Curr. Protoc. Bioinf. 25, 4.10.1–4.10.14. 10.1002/0471250953.bi0410s25 19274634

[B42] Vurture G. W. Sedlazeck F. J. Nattestad M. Underwood C. J. Fang H. Gurtowski J. (2017). GenomeScope: fast reference-free genome profiling from short reads. Bioinformatics 33, 2202–2204. 10.1093/bioinformatics/btx153 28369201 PMC5870704

[B43] Walker B. J. Abeel T. Shea T. Priest M. Abouelliel A. Sakthikumar S. (2014). Pilon: an integrated tool for comprehensive microbial variant detection and genome assembly improvement. PloS One 9, e112963. 10.1371/journal.pone.0112963 25409509 PMC4237348

[B44] Wences A. H. Schatz M. C. (2015). Metassembler: merging and optimizing de novo genome assemblies. Genome Biol. 16, 207. 10.1186/s13059-015-0764-4 26403281 PMC4581417

[B45] Winnepenninckx B. Backeljau T. De Wachter R. (1993). Extraction of high molecular weight DNA from molluscs. Trends Genet. 9, 407. 10.1016/0168-9525(93)90102-N 8122306

[B46] Wrange A.-L. Valero J. Harkestad L. S. Strand Ø. Lindegarth S. Christensen H. T. (2010). Massive settlements of the Pacific oyster, Crassostrea gigas, in Scandinavia. Biol. Invasions 12, 1145–1152. 10.1007/s10530-009-9535-z

[B47] Xiao C. L. Chen Y. Xie S. Q. Chen K. N. Wang Y. Han Y. (2017). MECAT: fast mapping, error correction, and de novo assembly for single-molecule sequencing reads. Nat. Methods 14, 1072. 10.1038/nmeth.4432 28945707

[B48] Xiao C. L. Zhu S. He M. Chen D. Zhang Q. Chen Y. (2018). N6-methyladenine DNA modification in the human genome. Mol. Cell 71, 306–318. 10.1016/j.molcel.2018.06.015 30017583

[B49] Xu Z. Wang H. (2007). LTR_FINDER: an efficient tool for the prediction of full-length LTR retrotransposons. Nucleic Acids Res. 35, W265–W268. 10.1093/nar/gkm286 17485477 PMC1933203

[B50] Yang Z. (2007). PAML 4: phylogenetic analysis by maximum likelihood. Mol. Biol. Evol. 24, 1586–1591. 10.1093/molbev/msm088 17483113

[B51] Zhang G. Fang X. Guo X. Li L. Luo R. Xu F. (2012). The oyster genome reveals stress adaptation and complexity of shell formation. Nature 490, 49–54. 10.1038/nature11413 22992520

[B52] Zhang G. Li L. Meng J. Qi H. Qu T. Xu F. (2016). Molecular Basis for Adaptation of Oysters to Stressful Marine Intertidal Environments. Annu. Rev. Anim. Biosci. 4, 357–381. 10.1146/annurev-animal-022114-110903 26515272

