# Supplementary material for: Nanopore Sequencing and De Novo Assembly of a Black-Shelled Pacific Oyster (Crassostrea gigas) Genome
Source: Front Genet. 2019 Nov 22;10:1211. doi: 10.3389/fgene.2019.01211 (PMC6884003; doi:10.3389/fgene.2019.01211)
Supplement: Supplementary file 1 [file DataSheet_1.docx]

Supplementary Material


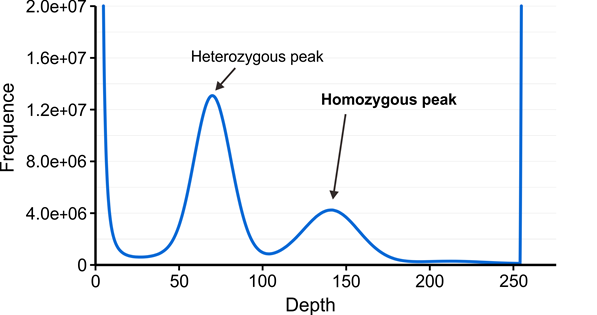


**Figure S1 23-mer frequency distribution in the black-shelled Pacific oyster.**


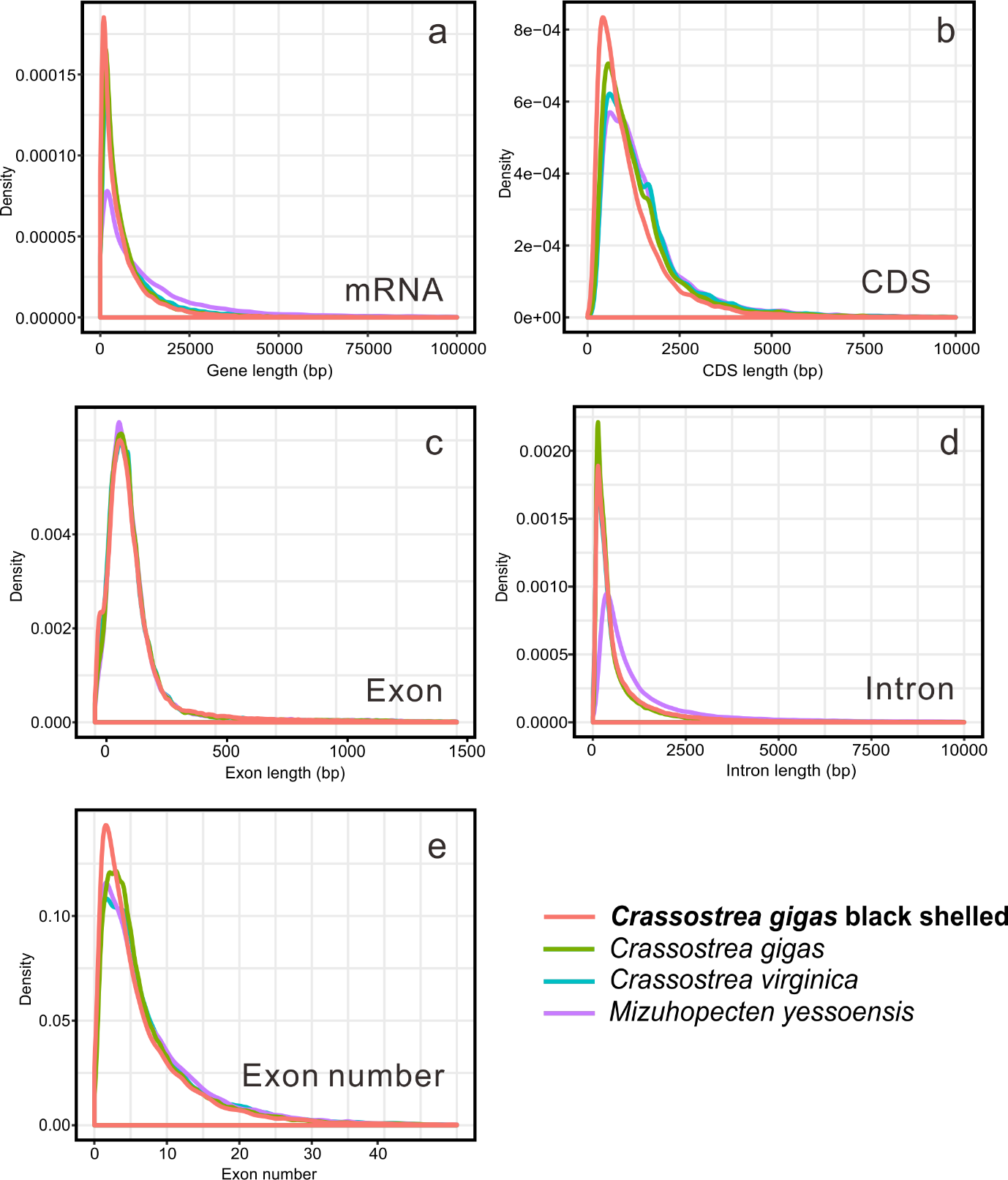


**Figure S2 Gene characteristics of black-shelled Pacific oyster and three other bivalve species used in gene prediction.** (a) mRNA length, (b) CDS length, (c) Exon length, (d) Intron length, (e) Exon number per gene.

**
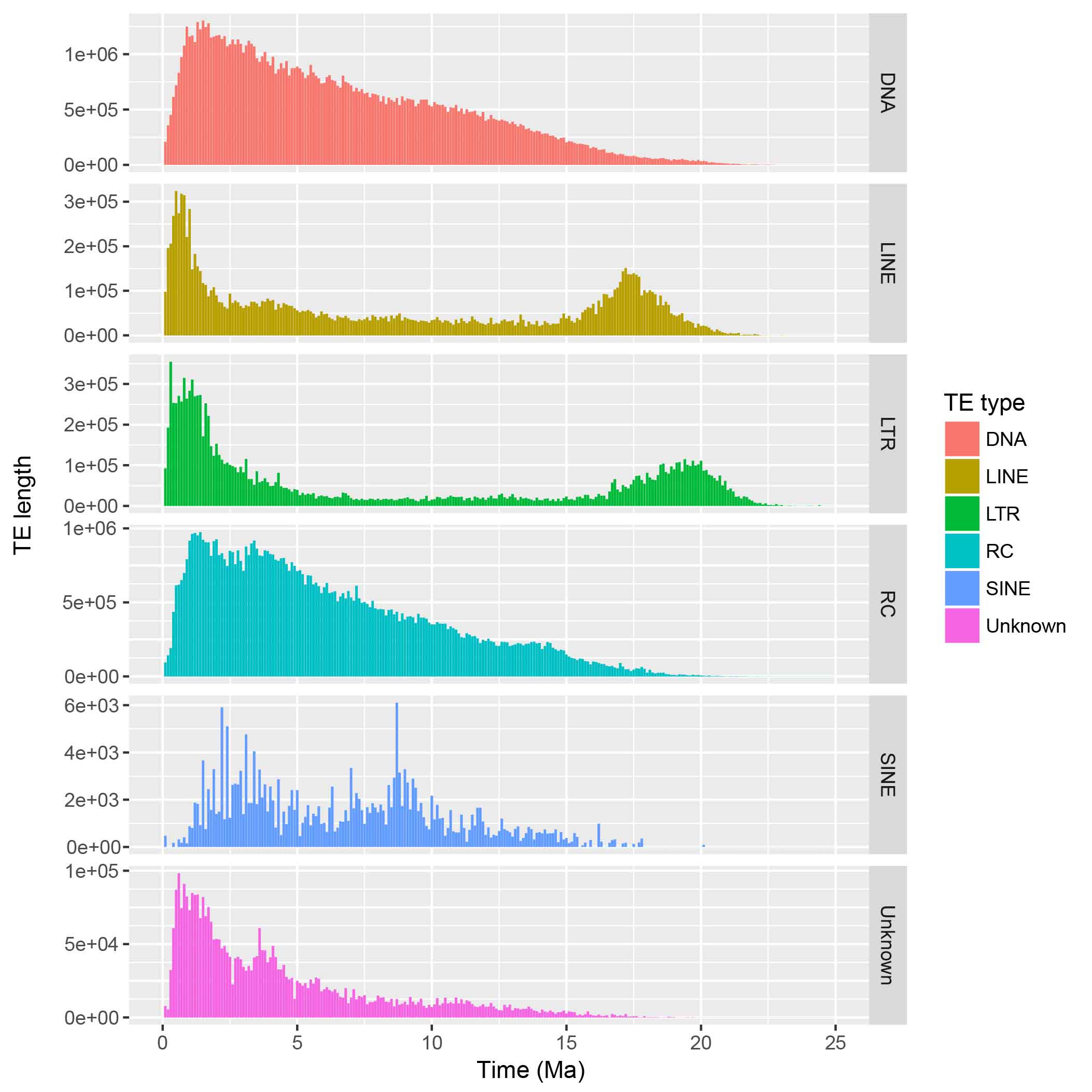
**

**Figure S3 Landscape of each kind of TE accumulation.** The x-axis is the Insertion time (Ma), and the y-axis is TE length.

**Table S1 Summary of sequencing data for black-shelled Pacific oyster.**

| Platform |  | MGISEQ-2000 | |  | GridION | |
| --- | --- | --- | --- | --- | --- | --- |
| Insert size (bp) |  | 150 bp | |  | 20kb | |
|  |  | Raw data | Filter data |  | Raw data | Corrected data |
| Number of bases (Gb) |  | 105.6 | 104.9 |  | 61.8 | 39.9 |
| Number of reads |  | 703,742,746 | 700,047,136 |  | 4,533,686 | 1,721,917 |
| Mean read length |  | 150bp | 149bp |  | 16.3Kb | 21.9Kb |
| Sequence coverage (X) |  | 177.7 | 176.7 |  | 104 | 67.2 |
| SRA accession |  | SRP193912 | — |  | SRP193912 | — |

**Table S2 Statistics of K-mer analysis.**

| K-mer | K-mer Number | Peak Depth | Genome Size | Used Bases | Used Reads | Sequence coverage (X) |
| --- | --- | --- | --- | --- | --- | --- |
| 23 | 83,754,003,275 | 141 | 594,000,023 | 104,958,363,642 | 700,041,696 | 176.7 |

**Table S3 Statistics of the assembly before and after** **Purge Haplotigs.**

| **Contigs** | **Before Purge Haplotigs** | | **After Purge Haplotigs** | |
| --- | --- | --- | --- | --- |
|  | **Length (bp)** | **Number** | **Length (bp)** | **Number** |
| N90 | 36,341 | 2,732 | 66,669 | 1,570 |
| N80 | 76,120 | 1,488 | 124,267 | 922 |
| N70 | 142,742 | 841 | 215,826 | 561 |
| N60 | 255,568 | 486 | 361,254 | 350 |
| N50 | 436,376 | 288 | 581,941 | 220 |
| Shortest | 3,203 | — | 3,241 | — |
| Longest | 6,082,460 | — | 6,082,460 | — |
| Total Size | 656,378,595 | 6,815 | 587,503,506 | 3,676 |
| Total Number (>10 Kbp) | — | 5,458 | — | 3,293 |
| Total Number (>100 Kbp) | — | 1,153 | — | 1,127 |
| Total Number (>1 Mbp) | — | 109 | — | 109 |

**Table S4 Statistics of mapping ratio of BGI-seq reads to assembled genome.**

| Total reads | Mapped reads | Mapped reads ratio | Properly paired | Properly paired ratio | genome coverage | Mean depth |
| --- | --- | --- | --- | --- | --- | --- |
| 713,784,840 | 695,357,884 | 97.42% | 639,954,950 | 91.42% | 98.08% | 164.71 |

**Table S5 Statistics for repetitive sequences identified in the black-shelled** **Pacific oyster genome classified according to the method of detection.**

| **Tool** | **Total repeat length (bp)** | **% of assembly** |
| --- | --- | --- |
| RepeatMasker | 221,326,498 | 37.67 |
| RepeatProteinMask | 18,546,488 | 3.16 |
| RepeatModeler | 253,930,788 | 43.22 |
| Tandem Repeats Finder | 33,441,376 | 5.69 |
| Combined | 283,873,692 | 48.32 |

**Table S6 Statistics for repetitive sequences identified in the black-shelled Pacific oyster genome classified according to the biological category.**

| **Category** | **Total repeat length (bp)** | **% of assembly** |
| --- | --- | --- |
| DNA | 125,006,742 | 21.28 |
| LINE | 17,773,051 | 3.03 |
| SINE | 828,517 | 0.15 |
| LTR | 17,932,709 | 3.05 |
| Other | 86,071 | 0.01 |
| Satellite | 619,081 | 0.11 |
| Unknown | 31,558,904 | 5.37 |
| Tandem repeat | 33,441,376 | 5.69 |
| Total | 283,873,692 | 48.32 |

**Table S7 Summary of protein-coding genes in black-shelled Pacific oyster.**

| **Total Genes** | **Average Gene Length (bp)** | **Average CDS Length (bp)** | **Average Exons per Gene** | **Average Exon Length (bp)** | **Average Intron Length (bp)** |
| --- | --- | --- | --- | --- | --- |
| 26811 | 6,762.5 | 1225.2 | 7 | 174.6 | 920.4 |

**Table S8 Statistics of gene function annotation of black-shelled Pacific oyster.**

|  | **Numbers of annotated genes** | **Percent (%) to all genes (26,811)** |
| --- | --- | --- |
| GO | 11,810 | 44.05 |
| InterProScan | 16,853 | 62.86 |
| PATHER | 15,997 | 59.67 |
| PFAM | 14,400 | 53.71 |
| KEGG | 7,575 | 28.25 |
| Number of annotated genes | 23,111 | 86.20 |
| Number of unannotated genes | 3,700 | 13.80 |

**Table S9** **GO enrichment expanded gene families in black-shelled Pacific oyster.**

| **GO** | **Type** | **Function** | **Chi.FDR** |
| --- | --- | --- | --- |
| GO:0016788 | molecular function | hydrolase activity, acting on ester bonds | 2.89E-59 |
| GO:0003676 | molecular function | nucleic acid binding | 5.75E-48 |
| GO:0004842 | molecular function | ubiquitin-protein transferase activity | 4.89E-26 |
| GO:0001614 | molecular function | purinergic nucleotide receptor activity | 2.03E-19 |
| GO:0004931 | molecular function | extracellularly ATP-gated cation channel activity | 2.03E-19 |
| GO:0033198 | biological process | response to ATP | 2.03E-19 |
| GO:0098655 | biological process | cation transmembrane transport | 2.03E-19 |
| GO:0008270 | molecular function | zinc ion binding | 7.31E-17 |
| GO:0004803 | molecular function | transposase activity | 1.88E-15 |
| GO:0051015 | molecular function | actin filament binding | 2.36E-15 |
| GO:0015074 | biological process | DNA integration | 5.01E-15 |
| GO:0006313 | biological process | transposition, DNA-mediated | 4.06E-12 |
| GO:0005887 | cellular component | integral component of plasma membrane | 6.44E-10 |
| GO:0016888 | molecular function | endodeoxyribonuclease activity, producing 5'-phosphomonoesters | 3.05E-08 |
| GO:0090286 | biological process | cytoskeletal anchoring at nuclear membrane | 1.53E-07 |
| GO:0003779 | molecular function | actin binding | 1.53E-06 |
| GO:0005635 | cellular component | nuclear envelope | 2.54E-06 |
| GO:0034993 | cellular component | meiotic nuclear membrane microtubule tethering complex | 2.54E-06 |
| GO:0005615 | cellular component | extracellular space | 8.72E-05 |
| GO:0000183 | biological process | chromatin silencing at rDNA | 0.0002 |
| GO:0005720 | cellular component | nuclear heterochromatin | 0.0002 |
| GO:0007156 | biological process | homophilic cell adhesion via plasma membrane adhesion molecules | 0.02 |

**Table S10 GO enrichment contracted gene families in black-shelled Pacific oyster.**

| **GO** | **Type** | **Function** | **Chi.FDR** |
| --- | --- | --- | --- |
| GO:0004601 | molecular function | peroxidase activity | 2.20E-27 |
| GO:0006979 | biological process | response to oxidative stress | 1.33E-21 |
| GO:0006950 | biological process | response to stress | 2.90E-21 |
| GO:0000786 | cellular component | nucleosome | 7.30E-19 |
| GO:0005507 | molecular function | copper ion binding | 9.56E-18 |
| GO:0007160 | biological process | cell-matrix adhesion | 2.60E-12 |
| GO:0009607 | biological process | response to biotic stimulus | 6.50E-10 |
| GO:0008146 | molecular function | sulfotransferase activity | 1.74E-09 |
| GO:1902751 | biological process | positive regulation of cell cycle G2/M phase transition | 8.13E-08 |
| GO:0004888 | molecular function | transmembrane signaling receptor activity | 6.00E-07 |
| GO:0006004 | biological process | fucose metabolic process | 9.33E-07 |
| GO:0070836 | biological process | caveola assembly | 9.33E-07 |
| GO:0008378 | molecular function | galactosyltransferase activity | 2.96E-06 |
| GO:0016614 | molecular function | oxidoreductase activity, acting on CH-OH group of donors | 5.41E-06 |
| GO:0022857 | molecular function | transmembrane transporter activity | 6.11E-06 |
| GO:0004560 | molecular function | alpha-L-fucosidase activity | 7.15E-06 |
| GO:0005524 | molecular function | ATP binding | 3.08E-05 |
| GO:0007166 | biological process | cell surface receptor signaling pathway | 5.41E-05 |
| GO:0003950 | molecular function | NAD+ ADP-ribosyltransferase activity | 0.0003 |
| GO:0006334 | biological process | nucleosome assembly | 0.001 |
| GO:0008745 | molecular function | N-acetylmuramoyl-L-alanine amidase activity | 0.001 |
| GO:0003676 | molecular function | nucleic acid binding | 0.002 |
| GO:0051607 | biological process | defense response to virus | 0.003 |
| GO:0046982 | molecular function | protein heterodimerization activity | 0.004 |
| GO:0005272 | molecular function | sodium channel activity | 0.005 |
| GO:0004866 | molecular function | endopeptidase inhibitor activity | 0.005 |
| GO:0009253 | biological process | peptidoglycan catabolic process | 0.006 |
| GO:0042834 | molecular function | peptidoglycan binding | 0.006 |
| GO:0005216 | molecular function | ion channel activity | 0.006 |
| GO:0006811 | biological process | ion transport | 0.01 |
| GO:0030374 | molecular function | nuclear receptor transcription coactivator activity | 0.04 |
